# Characterization of Transcription from TATA-Less Promoters: Identification of a New Core Promoter Element XCPE2 and Analysis of Factor Requirements

**DOI:** 10.1371/journal.pone.0005103

**Published:** 2009-04-01

**Authors:** Ramakrishnan Anish, Mohammad B. Hossain, Raymond H. Jacobson, Shinako Takada

**Affiliations:** Department of Biochemistry and Molecular Biology, Genes and Development Program of the Graduate School of Biomedical Sciences, The University of Texas M. D. Anderson Cancer Center, Houston, Texas, United States of America; University of Munich and Center of Integrated Protein Science, Germany

## Abstract

**Background:**

More than 80% of mammalian protein-coding genes are driven by TATA-less promoters which often show multiple transcriptional start sites (TSSs). However, little is known about the core promoter DNA sequences or mechanisms of transcriptional initiation for this class of promoters.

**Methodology/Principal Findings:**

Here we identify a new core promoter element XCPE2 (X core promoter element 2) (consensus sequence: A/C/G-C-C/T-C-G/A-T-T-G/A-C-C/A_+1_-C/T) that can direct specific transcription from the second TSS of hepatitis B virus X gene mRNA. XCPE2 sequences can also be found in human promoter regions and typically appear to drive one of the start sites within multiple TSS-containing TATA-less promoters. To gain insight into mechanisms of transcriptional initiation from this class of promoters, we examined requirements of several general transcription factors by *in vitro* transcription experiments using immunodepleted nuclear extracts and purified factors. Our results show that XCPE2-driven transcription uses at least TFIIB, either TFIID or free TBP, RNA polymerase II (RNA pol II) and the MED26-containing mediator complex but not Gcn5. Therefore, XCPE2-driven transcription can be carried out by a mechanism which differs from previously described TAF-dependent mechanisms for initiator (Inr)- or downstream promoter element (DPE)-containing promoters, the TBP- and SAGA (Spt-Ada-Gcn5-acetyltransferase)-dependent mechanism for yeast TATA-containing promoters, or the TFTC (TBP-free-TAF-containing complex)-dependent mechanism for certain Inr-containing TATA-less promoters. EMSA assays using XCPE2 promoter and purified factors further suggest that XCPE2 promoter recognition requires a set of factors different from those for TATA box, Inr, or DPE promoter recognition.

**Conclusions/Significance:**

We identified a new core promoter element XCPE2 that are found in multiple TSS-containing TATA-less promoters. Mechanisms of promoter recognition and transcriptional initiation for XCPE2-driven promoters appear different from previously shown mechanisms for classical promoters that show single “focused” TSSs. Our studies provide insight into novel mechanisms of RNA Pol II transcription from multiple TSS-containing TATA-less promoters.

## Introduction

Recent bioinformatics studies have revealed that most mammalian genes do not conform to the simple model in which a TATA box directs transcription from a single defined nucleotide position –most genes have multiple promoters, within which there are multiple start sites, and that 72% of human promoters are associated with CpG islands [1]–[3]. It has been also reported that the majority of strong human RNA polymerase II (RNA pol II) core promoters have an array of closely located transcriptional start sites (TSSs) that are spread over 50–100 bp [4], which is different from the traditional view that “true” or ”truly specific” transcriptional initiations show single (or “focused”) TSS. Broad TSS distributions (“dispersed” TSSs) are correlated with CpG islands and ubiquitously expressed genes, whereas promoters with a narrow TSS distribution frequently direct tissue-specific genes and often have a TATA box. [5]. The frequency of TATA box containing promoters among human protein-coding genes is now estimated to be 10–20% [2], [3], [6].

Ironically, most of the studies examining fundamental mechanisms of transcriptional regulation have been carried out using promoters that have “focused” start sites, particularly, TATA-containing promoters. Thus, how the transcriptional machinery recognizes “dispersed” promoters and initiates transcription from multiple, individual TSSs (or “TS regions”) is still poorly characterized. A number of questions remain regarding the mechanisms at play for promoters utilizing multiple start sites; (1) whether individual start sites are specifically driven by definitive core promoter elements or whether a single “loose” element can drive transcription from multiple locations, (2) how transcription from different start sites within a promoter can be differently regulated, (3) which general transcription factors (GTFs) are used for transcription from different start sites at these types of promoters, and (4) whether a stable preinitiation complex is formed for transcriptional initiation from each start site. (5) Previously identified core promoter elements including the TATA box, the initiator (Inr), the downstream promoter element (DPE), the TFIIB recognition element (BRE), the motif ten element (MTE), downstream core element (DCE), and XCPE1 [6]–[11] are not present in a large number of genes in the mammalian genome [6], [7], [12], [13]. Are there other as yet unidentified sequences responsible for transcriptional activity? To address these questions, we must identify which DNA sequences (i.e., core promoter elements) are essential to drive transcription from each TSS, examine the properties of these newly identified core promoters, and subsequently determine GTF and cofactor requirements for these newly identified promoters.

In this report, using extensive mutagenesis we identify a novel DNA sequence that functions as a core promoter element at the second start site of the hepatitis B virus (HBV) X mRNA which we have named XCPE2 (X core promoter element 2). The core promoter containing XCPE2 is located in one of the CpG islands of the HBV genome in a similar way to observed TSSs in the human genome. Our search of promoter database shows that XCPE2 like sequences also appear to be present in human TATA-less promoters and typically drive one of the start sites present within multiple TSS-containing promoters. Our *in vitro* transcription analyses using immunodepleted nuclear extracts with purified GTFs show that XCPE2-driven basal transcription requires at least RNA pol II, TFIIB, MED26-containing mediator, and either a free form of TBP or TFIID. We further show in this report that XCPE2-driven transcription is Gcn5-independent and therefore is independent of TFTC [14] or STAGA [Spt3-TAF_II_31-Gcn5-L-acetyltransferase] [15] (human homologue of yeast SAGA) complex. We also observed in our *in vitro* transcription analyses with cellular XCPE2-containing promoters that transcription from not only the XCPE2-driven TSSs but also other TSSs in the XCPE2-containing promoter regions show similar GTF requirements to those of the X gene promoter. Our study on XCPE2-driven transcription also provides, in our knowledge, the first example that clearly demonstrates mediator-dependent basal (but not activated) transcription from TATA-less promoters. This property may potentially be applied to many other promoters localized in CpG islands. Our studies provide essential information to help understand mechanisms of RNA Pol II transcription from dispersed promoters.

## Results

### Determination of the core promoter element for transcription from Start site 2 of the HBV X mRNA

**Figure 1:**
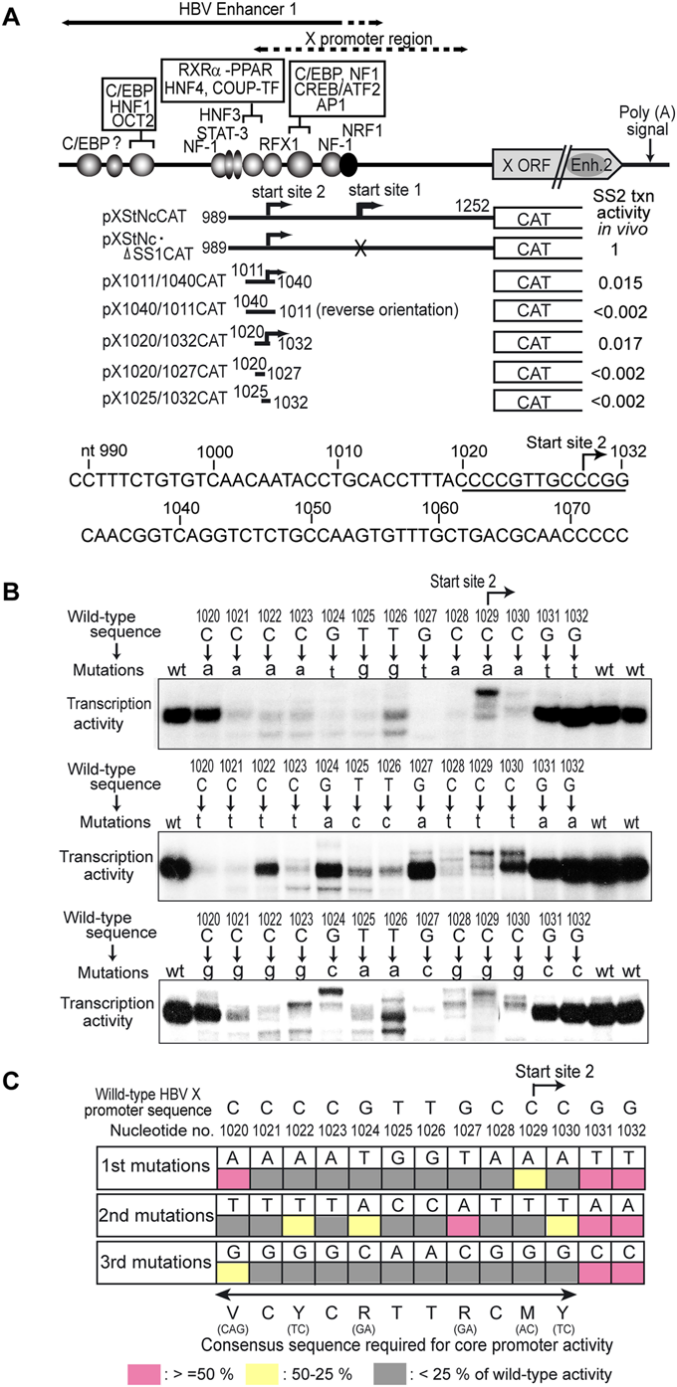
Determination of the core promoter element driving transcription from HBV X mRNA Start site 2. (A) Schematic of the HBV enhancer 1-X promoter region and the deletion mutants used for mapping of the minimal promoter for transcription from Start site 2. Different HBV enhancer 1-X promoter fragments (nucleotide numbers of the boundaries shown) were cloned into a CAT reporter plasmid and used as the template for *in vitro* transcription. Summary of the *in vitro* transcription analyses are schematically shown. The bent arrows show that accurate transcription from Start site 2 [6], [47] was detected with the indicated constructs. Being the same thicknesses of the bent arrows indicate about the levels of transcription detected by *in vitro* transcription assays. Absence of the bent arrows on the promoter region indicates no detectable transcription from the particular DNA fragments in the right direction. On the right ends, relative transcription activity of each construct *in vivo* (in transfected HepG2 cells) measured by CAT assay is shown as “SS2 txn activity *in vivo*”. The template pXStNc·ΔSS1CAT has mutation at the Start site 1 core promoter so that only transcription from Start site 2 can be measured. The DNA sequence around the Start site 2 minimal promoter region is shown at the bottom. The 13-bp minimal promoter region is underlined and the position of Start site 2 is shown by a bent arrow. (B) *In vitro* transcription assays of the wild-type and point-mutated minimal promoters for the Start site 2. The nucleotides within the 13-bp minimal promoter region were individually mutated into three other nucleotides, and the transcription activity of the mutants was assayed *in vitro*. Primer extension products corresponding to Start site 2 are shown. The levels of transcription from mutant templates were quantified by phosphor-imager analysis. (C) Summary of site-directed mutagenesis. The *in vitro* transcription assays were repeated at least three times with several independent preparations of template DNAs, and average activity of each mutant relative to that of the wild-type minimal promoter was calculated. Promoter activities of mutants are categorized into three groups based on their relative activity to the wild-type promoter: pink, ≥50%; yellow, 50–25%; gray, <25%. The consensus sequence deduced from our analysis is shown at the bottom of the figure.

We previously localized the core promoter activity for transcription from Start site 2 of the HBV X mRNA to a 13-bp DNA region between nt1020 and 1032 (CCCCGTTGCC_+1_CGG) that was located between −9 to +4 relative to Start site 2 (Fig. 1A, pX1020/1032CAT and [6]). This 13-bp DNA fragment could drive unidirectional transcription from the specific start site either in the context of HBV DNA or by itself when cloned into a reporter plasmid. Transcription activity of this 13-bp minimal promoter is about the same as Start site 2 transcription activity of the enhancer-containing constructs when measured by the *in vitro* transcription assays using naked DNA templates. However, it is lower than the Start site 2 transcription activity of the enhancer-containing constructs when measured in HepG2 cells (Fig. 1A, shown as “SS2 txn activity *in vivo*”), indicating that transcription from Start site 2 has an ability to be activated by enhancer elements. This 13-bp DNA sequence does not correspond to any of the previously described core promoter elements, nor does the region surrounding the 13-bp sequence contain any other previously known core promoter elements that can explain transcriptional initiation from Start site 2 (Fig. 1A, bottom). The 13-bp sequence also does not contain any complete previously known transcription activator-binding sites. Therefore, to determine what specific DNA sequence(s) within this region were critical for the promoter activity, we carried out extensive mutagenesis of the 13-bp sequence. We generated the complete set of single nucleotide point mutants and tested their transcription activity. Fig. 1B shows representative results of the *in vitro* transcription assays using the wild-type and mutant templates. Fig. 1C summarizes the results of the transcription assays of the 39 mutant promoters. The nucleotides critical for the core promoter activity were located in the 11-bp region between nt1020 and nt1030. The functionally tolerant range of a single base change from the HBV sequence (i.e., the sequence that showed higher than 25% of the wild-type promoter activity) was VCYCRTTRCMY.

### The core promoter element XCPE2 also drives transcription from promoters in the human genome

Since HBV genes are transcribed by the host RNA pol II, we were curious to see if XCPE2 is also utilized in human gene promoters. A human promoter database DBTSS (Version 6.0; http://dbtss.hgc.jp/, covering 15,262 genes in the human genome) was searched for the XCPE2 consensus sequence. Since most human genes have multiple promoters, individual promoters of such genes have their own entries in DBTSS that can be individually searched for sequence motifs. They are termed alternative promoters AP1, AP2, etc. However, within individual promoters, there are often multiple TSSs that show broad distribution. For those promoters, one of the start sites has been picked up to represent the promoter. Therefore, we first searched relatively broad regions of promoters (between the nucleotides −400 and +200 relative to the representative start sites) for the XCPE2 consensus sequence. This search identified 297 genes that contained XCPE2 sequences in the specified regions. Supplemental Table S1 shows a short list of the identified genes that have one or more previously mapped start sites within regions +/−20 bp from the XCPE2 sequences. The candidate human promoters appeared either typically to have an array of multiple start sites that had been detected at different frequencies, or to be the promoters for which start sites were still poorly mapped (Table S1). In the candidate promoters, we could find a number of start sites that occurred exactly at or near the positions expected to be driven by XCPE2. It is possible that more XCPE2-driven start sites will be identified as the determination of TSSs for the database becomes more complete. However, to fairly determine whether the XCPE2 sequences found in these promoters were actually functional, it seemed necessary to analyze TSSs of individual promoters by direct analyses of RNA transcripts such as primer extension analysis. Direct analysis of TSSs has some advantages over searching a promoter database when analyzing promoters that are present in CpG islands, because of the way the promoter database has been made: DBTSS was made using the information on 5′ ends of cDNA clones present in cDNA libraries that were constructed through a process involving reverse transcription, PCR, cloning to a plasmid, and amplification of the library. It is known that highly GC-rich DNAs are often difficult to be PCR amplified, are easily structurally rearranged during cloning, and tend to produce poor sequence results. Moreover, rearranged cDNA clones tend to be amplified more efficiently than original clones during cDNA library amplification [16], [17]. Therefore, TSSs in CpG islands are potentially incompletely represented in the database.

**Figure 2:**
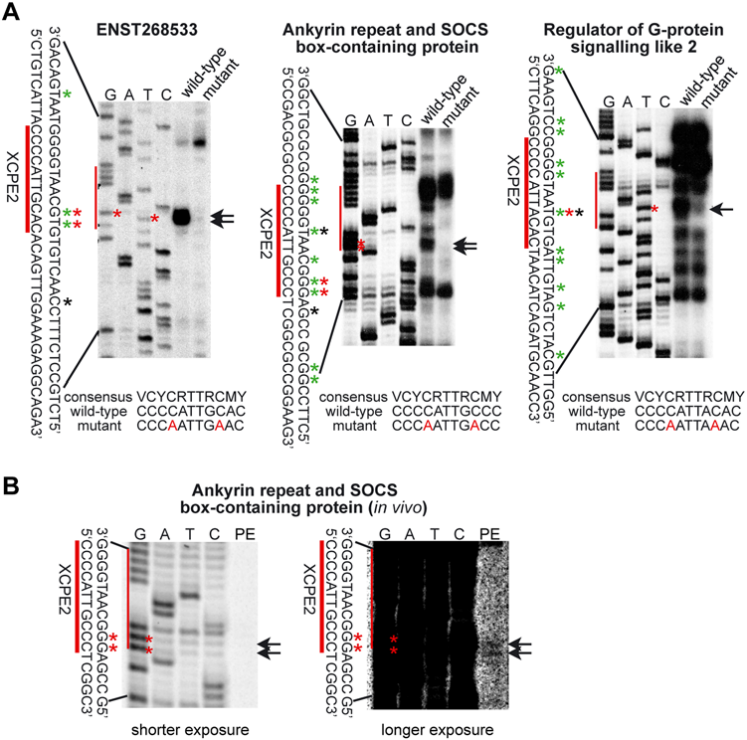
XCPE2 drives transcription from human promoters. (A) *In vitro* transcription analyses of wild-type and XCPE2-mutated promoter templates. Sequence ladders were made using the same sets of templates and primers as those for the primer extension analyses. Arrows and red asterisks (*) show the TSSs at the position expected to be driven by XCPE2. Green asterisks show the nucleotide positions of other start sites detected in our *in vitro* transcription assays. Black asterisks show the nucleotide positions of start sites recorded in the DBTSS database. (B) Primer extension analysis of Ankyrin repeat and SOCS box-containing protein mRNA produced in transfectecd HepG2 cells, showing that the same TSSs driven by XCPE2 as *in vitro* transcription assays were detected. Arrows and red asterisks show the TSSs driven by XCPE2.

To directly identify TSSs of cellular promoters, we cloned several candidate XCPE2-containing human promoters and conducted *in vitro* transcription assays. As shown in Fig. 2A and summarized in Table S1, we were able to detect TSSs at the positions expected to be driven by XCPE2 in these promoters. For example, the TSS of “Regulator of G-protein signaling like 2” reported in DBTSS (shown by black asterisk in Fig. 2A and Table S1) exactly matched the position expected to be driven by XCPE2 (shown by red asterisk in Fig. 2A) and one of the TSSs detected by our *in vitro* transcription assay (shown by green asterisks in Fig. 2A, and by red asterisks in Table S1). Even in the case of “Ankyrin repeat and SOCS box-containing protein” and ENST00000268533 for which no start sites had yet been mapped at XCPE2 in DBTSS, we could detect the start sites at the positions expected if driven by the XCPE2 sequences. The XCPE2 start site in the “Regulator of G-protein signaling like 2” promoter appeared shifted slightly (2 or 3 bp) upstream from the usual +1 site for XCPE2, but that may be explained by the observation shown in Fig. 1B that nucleotide exchange at the +1 position (nt1029 of HBV sequence) appeared to influence start site selection. In our previous study of XCPE1 core promoter consensus sequence, we also observed minor start site shifts when the consensus sequence diverged from the original XCPE1 sequence in the HBV DNA [6].

Since the XCPE2-driven TSS in the promoter of “Ankyrin repeat and SOCS box-containing protein” has not been recorded in DBTSS, we additionally analyzed transcripts from this promoter produced *in vivo*, i.e., in transiently transfected cells, by primer extension (Fig. 2B). Since the promoter region we cloned (−480 to +46 relative to the expected TSS driven by the XCPE2) did not necessarily contain whole promoter (and enhancer) of this gene, the level of transcription was very low, but we were able to detect TSSs at the position expected to be driven by XCPE2 (shown by arrows in Fig. 2B). The level of transcription of ENST268533 was below the detection limit in our transiently transfection & primer extension experiments under the conditions used, likely due to lack of enhancer in our promoter construct. However, we have observed consistent TSS selection patterns between *in vivo* transcription and our *in vitro* transcription for all of the more than 15 gene promoters we have analyzed (Tokusumi et al. (2007), this study, and unpublished results).

To further examine if the XCPE2 sequences in these promoters are functioning as core promoter elements, we mutated the XCPE2 sequences and tested the effect of the mutations on the promoter activity. Strikingly, the XCPE2 mutations specifically abolished transcription from the start sites at the position expected to be driven by XCPE2 with minimal influence to other neighboring start sites (Fig. 2A). It should be noted that there is a sequence TTACACT in the promoter of “Regulator of G-protein signaling like 2” which matches the Inr consensus sequence YYA^+1^NWYY, partially overlapping with later half of the XCPE2 sequence CCCCATTACAC. However, this Inr sequence was found to be non-functional in this context because our mutation (CCCCATTACACT to CCCAATTAAACT) which changed the XCPE2 sequence into a non-XCPE2 sequence kept the Inr sequence within its consensus but resulted in a large transcriptional inhibition from the expected TSS. Thus, transcription from the start site expected to be driven by XCPE2 appears to be indeed driven by XCPE2 but not by the Inr sequence. These results verify that XCPE2 is indeed used for transcription from the specific sites in these human promoters. To determine what DNA sequences are important for transcription from each of other start sites in these promoters, extensive mutagenesis of individual promoters will be required. However, our results concerning XCPE2 sequences suggest that the other individual TSSs may be driven specifically by their each independent core promoter elements but not by XCPE2. In this sense, promoter recognition by the transcriptional machinery appears specific but the machinery may recognize a number of different sequences as core promoter elements.

### Examination of TFIID requirement for transcription from XCPE2-containing promoters

**Figure 3:**
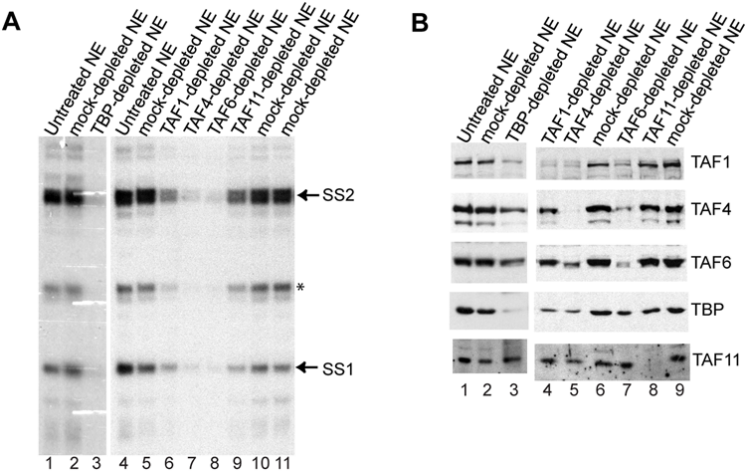
TFIID present in nuclear extracts contributes in X mRNA transcription, but TAF11 is dispensable. (A) *In vitro* transcription assays of immmunodepleted nuclear extracts (NEs). To examine importance of TFIID for X gene transcription, TFIID was depleted from NEs using antibodies raised against different TFIID subunits and the depleted NEs were tested for X gene transcription activity using the HBV enhancer-X promoter construct. SS1 and SS2: primer extension products showing transcription from Start site 1 and Start site 2, respectively. The asterisk shows a primer extension product that was not consistently observed. (B) Western blot analyses of the immunodepleted NEs. Depleted NEs were examined for the levels of TAF1, TAF4, TAF6, TAF11, and TBP.

To gain insight into mechanisms of transcriptional initiation from this class of promoters, we started investigating which general transcription factors are required. Among the known GTFs, TFIID has been shown to play a central role in promoter recognition for not only TATA box-containing promoters but also of DPE-, Inr-, or DCE-containing promoters. TFIID consists of TBP and about 15 TAF subunits. TBP recognizes TATA box; TAF6 and TAF9 recognize DPE; TAF1, TAF2, and TBP together recognize a class of Inr; and TAF1 recognizes DCE [7], [11], [18], [19]. Therefore, we first examined requirements for the TFIID complex. To analyze TFIID, it is important to keep in mind that there are other transcription factors that share some subunits with TFIID. For example, STAGA contains TAF5, 6, 9, 10, and 12 [20] and TFTC contains TAF2, 4, 5, 6, 7, 9, 10, and 12 [21]. TBP is also known to be a component of B-TFIID, TFIIIB, and SL1. Furthermore, a recent report suggested TFIID can have a stable core sub-complex (consisting of TAF4, 5, 6, 9, and 12) [22]. Therefore, to evaluate contribution of TFIID to XCPE2-driven transcription, we treated crude nuclear extracts (NEs) with antibodies against several TFIID subunits, including TFIID-specific subunits (TAF1 and TAF11) and the subunits shared by other factors (TAF4, and TAF6, and TBP). Then, we carried out *in vitro* transcription assays using the immunodepleted NEs. As shown in Fig. 3A, depletion of the tested TAFs and TBP all reduced transcription activity to some extent, indicating that TAF1, 4, 6, 11, and TBP were all involved in the transcription from the X gene Start site 2. However, it was interesting that depletion of the shared subunits TAF4, TAF6, and TBP showed a severer transcriptional reduction than that of the TFIID-specific subunits TAF1 or TAF11. In these experiments, we used the whole enhancer 1/X promoter template pBS-HBXB [6] so that we could also monitor transcription from Start site 1. We found that transcription from start sites 1 showed similar responses to the immunodepletion.

To help understand why TAF1- and TAF11-depletion showed weaker transcriptional inhibition than TAF4-, TAF6-, or TBP-depletion, we examined the protein levels of these subunits after the immunodepletion (Fig. 3B). We found that all of the TFIID antibodies used here (anti-TAF1, anti-TAF4, anti-TAF6, anti-TAF11, and anti-TBP) largely reduced the levels of their own target antigens, but also relatively efficiently co-depleted (reduced) all of the other TFIID subunits tested except TAF11 (Fig. 3B). These co-depletions were consistent with previous observations by others that these subunits are part of the TFIID complex. Previous observations have also suggested TAF11 may be less tightly associated with the rest of the TFIID subunits than TAF1, 4, 6, and TBP do [19], [22]. Therefore, the weak transcriptional inhibition by anti-TAF11 treatment we observed is likely to reflect a weak co-depletion of other TFIID components. The observation that efficient depletion of TAF11 itself did not result in a loss of transcriptional activity indicates that TAF11 may not be a critical component of TFIID for X gene transcription.

The weak transcriptional inhibition by TAF1-depletion may be a reflection of multiple pathways for X mRNA transcription that could include TAF1-independent transcription using free TBP or an incomplete (TAF1-free) TFIID complex etc. However, it may also be due to relative incompleteness of TFIID depletion by the anti-TAF1 in this particular experiment, because in the immunodepletion shown in a later figure of this report where the NE was more extensively depleted of TAF1 by treating with anti-TAF1 twice, we observed stronger transcriptional inhibition and co-depletion (reduction) of TAF4 and TAF6.

### Free TBP, instead of the complete TFIID complex, could drive X gene transcription without cooperation with TAFs

Nevertheless, we wanted to make clear whether free TBP could drive transcription from XCPE2 promoters, with the following reasons. [1] Since depletion with anti-TAF4, anti-TBP, and TAF6 inhibited X gene transcription more efficiently than depletion with anti-TAF1 in the experiments shown in Fig. 3, there was a possibility that free TBP might be able to function in conjunction with some TAF1-free TAF4/TAF6-containing complexes (i.e., TFIID sub-complex, STAGA, or TFTC). [2] In our previous study examining transcription from Start site 1 of the HBV X gene, we found that either free TBP or the complete TFIID complex could restore XCPE1-driven transcription to TBP-depleted NE. We were curious to see whether it is also the case for XCPE2-driven transcription. [3] In yeast cells, a free form of TBP exists and works with SAGA complex to drive TATA-containing promoters [23].

**Figure 4:**
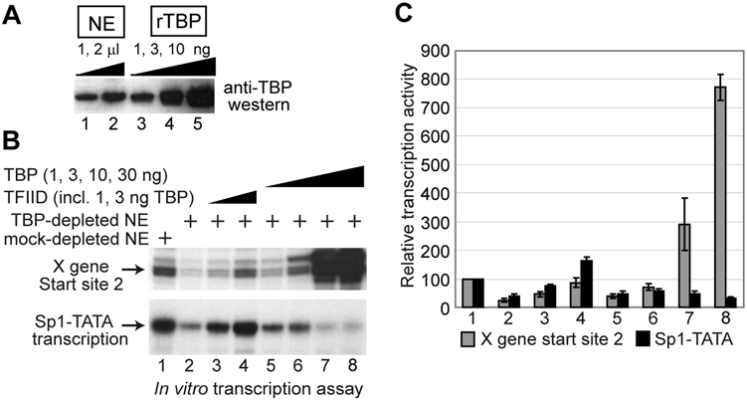
Transcription from X mRNA Start site 2 can use either a free TBP or the TFIID complex. (A) Examination of TBP concentration in HeLa NE. Indicated amounts of HeLa NE and purified recombinant TBP were loaded on a SDS-PAGE gel, and TBP was detected by anti-TBP western blotting. (B) *In vitro* transcription assays of the X gene (from Start site 2) and the Sp1-TATA templates (in a single two-template reaction). Control (lane 1) or TBP-depleted (lanes 2–8) NEs were tested for X gene or Sp1-TATA transcription in the absence (lane 2) or presence of 1 µl (lane 3) or 3 µl (lane 4) of purified TFIID or the presence of 1 ng (lane 5), 3 ng (lane 6), 10 ng (lane 7), or 30 ng (lane 8) of purified recombinant TBP (1 µl of the TFIID used in this experiment contained about 1 ng of TBP [6]). The enhancer-X promoter template was used to measure transcription from Start site 2. (C) Quantification results of the transcription assay. Transcription assays were performed twice for the reaction with 30 ng TBP (lane 8 of panel A) and four times for all of the other reactions. Brackets show standard errors of means.

In order to determine whether free TBP could drive transcription form XCPE2 promoters, we carried out an *in vitro* TBP add-back experiment. We first estimated how much TBP was present in the NE by western blotting. Since 1 µl of our NE showed about the same intensity of signal as 1 ng of purified recombinant TBP (Fig. 4A), 7.5 µl of NE which was going to be used for the *in vitro* transcription assays was estimated to contain about 7.5 ng of TBP. Therefore, we used 1, 3, 10, and 30 ng of purified TBP for the *in vitro* add-back experiments (Fig. 4B and C, lanes 5–8). As a control, we used purified TFIID that contained 1 or 3 ng of TBP (Fig. 4B and C, lanes 3 and 4). We found that free TBP could restore the X gene Start site 2 transcription activity to the TBP-depleted NE almost as efficiently as purified TFIID. This result suggests that transcription from the X promoter Start site 2, like Start site 1 [6], could use either a free form of TBP or the TFIID complex. When higher levels of TBP (10 ng and 30 ng) were added, X gene transcription was further activated. As a control, we monitored transcription from Sp1-TATA promoter, which shows activated transcription in a TFIID-dependent manner [24]. Transcription from Sp1-TATA was much more efficiently restored by TFIID than TBP, indicating that our TFIID was functional. The higher doses of free TBP rather inhibited Sp1-TATA transcription probably by competitively inhibiting TFIID binding to the TATA box. We saw an extra band several base-pair upstream of Start site 2 which became much stronger than original when high concentrations of free TBP were added. This band probably reflects transcriptional initiation induced by TBP-binding to the upstream region (perhaps the sequence AACAATA that is similar to TATA box) with loose sequence specificity. This phenomenon was not observed when transcription assay was carried out with the Start site 2 minimal promoter (Supplemental Fig. S1), therefore, the transcripts corresponding to this extra band are not likely to be driven by XCPE2.

Our results indicated that TFIID in NEs contribute to the *in vitro* X gene transcription reactions, but if a free form of TBP is available, free TBP can also promote efficient transcription (Figs. 3 and 4).

**Figure 5:**
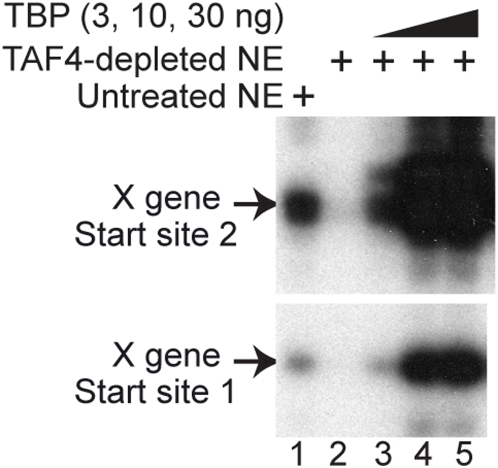
A free form of TBP can drive X gene transcription in the absence of TAF4. HeLa NE was depleted with nothing (lane 1) or with anti-TAF4 (lanes 2–5) and was mixed with the indicated amounts of purified recombinant TBP (lanes 3–5), then tested for X gene transcription activity.

However, it was still unclear whether free TBP promotes X gene transcription through cooperation with TAFs (i.e., in conjunction with the core TFIID sub-complex, STAGA, or TFTC) or without TAFs. To make this point clear, we tested if free TBP could restore X gene transcription activity to the TAF4-depleted NE by an *in vitro* add-back experiment. As shown in Fig. 5, we found that the free TBP could promote X gene transcription independently of TAF4, suggesting the core TFIID sub-complex and other TAF4-containing complexes such as TFTC or STAGA are not necessary for XCPE2 transcription with free TBP. The TFTC- or STAGA-independence of X gene transcription was further demonstrated by the Gcn5-depletion experiment shown later. Therefore, the X gene transcription with free TBP is carried out through a mechanism different from that with TBP+SAGA for yeast TATA-dependent transcription or the mechanism with TFTC for the Inr-containing TATA-less transcription of TEF-1 (transcription-enhancer factor-1) gene [14].

**Figure 6:**
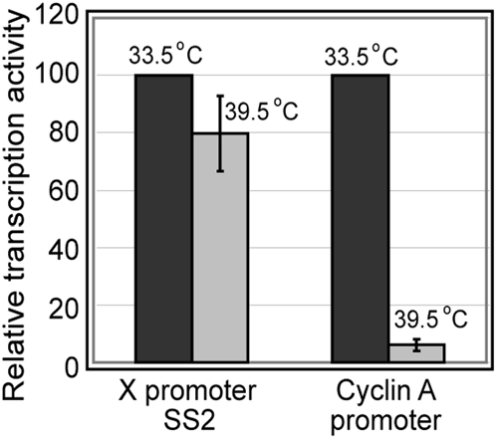
TAF1 is marginally important for X gene transcription in ts13 cells. ts13 cells were transfected with a firefly luciferase reporter plasmid driven by the X gene core promoter 2 or the cyclin A promoter. After 16 hr of incubation at the permissive (33.5°C) or nonpermissive (39.5°C) temperature, the luciferase activity in transfected cells were measured and normalized for transfection efficiency. Brackets show standard error of means.

We also wanted to examine the role of TAF1 in XCPE2 transcription *in vivo*. We used a ts13 cell line that has a TAF1 missense mutation [25] in its potential HAT (histone acetyltransferase) region, and shows TAF1-defective phenotype without changing overall structure of TFIID at the non-permissive temperature [26]. The ts13 cells were transfected with a reporter plasmid driven by XCPE2 and cultured at either 33.5°C (permissive temperature) or 39.5°C (non-permissive temperature) (Fig. 6). As a control, transcription from TAF1-dependent cyclin A promoter [27] was analyzed in parallel. As expected, the level of cyclin A transcription was reduced about 15-fold upon inactivation of TAF1 at 39.5°C. Under the same experimental conditions, transcription by XCPE2 was only marginally decreased (about 1.3-fold) (Fig. 6). These results suggest that, in the absence of functional TAF1, ts13 cells could use an alternative mechanism for X gene transcription, i.e., a TAF1-independent mechanism either using free TBP or a functionally incomplete (TAF1-inactive) TFIID complex. This observation is consistent with the notion we obtained from *in vitro* studies that XCPE2-containing promoters can utilize either the complete TFIID complex or free TBP.

### Mediator and TFIIB but not Gcn5 are required for X gene transcription

**Figure 7:**
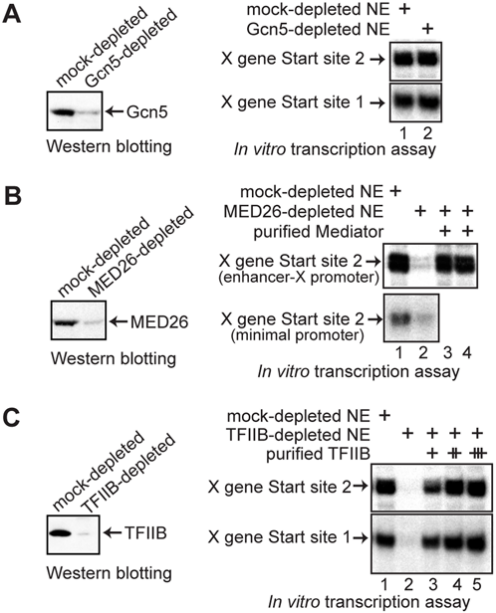
Transcription from X mRNA Start site 2 requires MED26-containing mediator and TFIIB but not Gcn5. (A) HeLa cell nuclear extract (NE) was immunodepleted with control or anti-Gcn5 antibody. The depleted NEs were then tested for the level of depletion by western blotting and for X gene transcription activity from the two start sites using the enhancer-X promoter construct. (B) HeLa NE was immunodepleted with control or anti-MED26 antibody. The depleted NEs were then tested for the level of depletion and for X gene Start site 2 transcription activity using either the enhancer-X promoter construct or the minimal promoter construct in the absence (lane 2) or presence (lanes 3 and 4) of mediator complexes purified from P.5 (lane 3) or P1.0 (lane 4) phosphocellulose fractions. The response of the transcription from Start site 1 has been reported [6]. (C) HeLa NE was immunodepleted with control or anti-TFIIB antibody. The depleted NEs were then tested for depletion and for X gene transcription activity from the two start sites using the enhancer-X promoter construct in the absence (lane 2) or presence (lanes 3–5) of the purified TFIIB (10, 30, or 100 ng).

The TFTC complex has been shown to contain subunits of the core TFIID sub-complex, and to be able to drive transcription from both TATA-containing and TATA-less Inr-containing promoters [14]. To examine if TFTC is involved in the transcription of the X gene, we examined the requirement for Gcn5, the major subunit of TFTC. As shown in Fig. 7A, we found that immunodepletion of Gcn5 did not change the X gene transcription activity of the NE, suggesting that TFTC is not required.

We have observed that anti-TBP, but not our anti-TAF1, could co-precipitate the MED26-containing mediator complex, thus TBP plus mediator might play an important role in X gene transcription. As shown in Fig. 7B, we found that treatment of NEs with anti-MED26 abolished transcription, suggesting that the MED26-containing mediator complex is required for X gene transcription. To further test the mediator requirement, we purified MED26-containing complexes and asked whether the purified MED26-containing complex restores X gene transcription activity to the MED26-depleted NE. Since it has been reported that the mediator complexes from different phosphocellulose fractions (0.5 M or 0.85 M KCl eluates) might have different roles in transcriptional control [28], [29], we purified MED26-containing complexes from both 0.5 M and 1 M KCl phosphocellulose fractions (P.5 and P1.0) and tested their activity. The add-back experiment further supported the idea that the MED26-containing mediator complex is required for X gene transcription and showed that the MED26 complexes from either phosphocellulose fractions could support X gene transcription. We also carried out this immunodepletion experiment using the minimal promoter construct (pX1020-1032CAT shown in Fig. 1A) and obtained essentially the same result (Fig. 7B lower panel). Since the minimal promoter region does not contain any sequence-specific DNA-binding activator binding sites, the result shows that the mediator is required for “basal” transcription from Start site 2.

We also examined the requirement for TFIIB by an immunodepletion and add-back experiment, and found that TFIIB was required for X gene transcription (Fig. 7C).

### X gene transcription with free TBP requires cooperation with TFIIB, RNA pol II and the MED26-containing mediator

**Figure 8:**
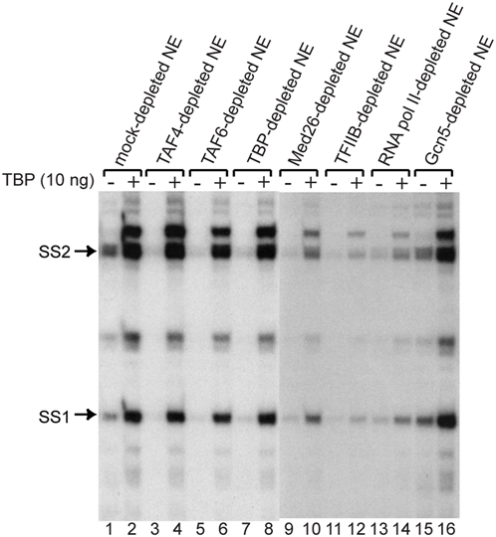
X gene transcription with free TBP requires mediator, TFIIB, and RNA pol II but not TAFs or Gcn5. HeLa NE was immunodepleted of the indicated factors and tested for X gene transcription activity with or without addition of free TBP. Abolishment of X gene transcription by TAF4-, TAF6-, TBP-, MED26-, TFIIB-, or RNA pol II-depletion (lane 1 vs. lanes 3, 5, 7, 9, 11, and 13) confirms that these factors contribute to the X gene transcription but Gcn5 doesn't (lane 1 vs. lane 15). The strong activation of X gene transcription by addition of a higher-than-endogenous level of free TBP to the mock-, TAF4-, TAF6-, TBP-, and Gcn5-depleted NEs (lanes 2, 4, 6, 8, and 16) indicates that these depleted factors were not necessary for X gene transcription by the mechanism using free TBP. In contrast, the absence of such activation by addition of free TBP to MED26-, TFIIB-, or RNA pol II-depleted NE (lanes 10, 12, and 14) indicates that these three factors are required for the free TBP-driven transcription to occur. For more details, see text.

Since we found that free TBP could activate X gene transcription independently of TAF4, we next asked what other factors are necessary for the TAF-free XCPE1- or XCPE2-driven transcription. For this purpose, we added free TBP to various immunodepleted NEs. As shown in Fig. 8, free TBP could activate X gene transcription without TAF4, TAF6, or Gcn5 to a higher level than observed for the untreated NE. However, without MED26, RNA pol II, or TFIIB, activation by free TBP was largely compromised, indicating that the mediator, RNA pol II, and TFIIB have important roles separate from that of free TBP. The small increases of transcription by TBP addition in the MED26-, TFIIB-, or RNA pol II-depleted NE (Fig. 8, lanes 10, 12, and 14 relative to lanes 9, 11, and 13) were most likely the result of transcription carried out by residual mediator, RNA pol II, or TFIIB due to incomplete immunodepletion.

### XCPE1 and XCPE2-containing cellular promoters show the same GTF requirements as the HBV X gene promoter

**Figure 9:**
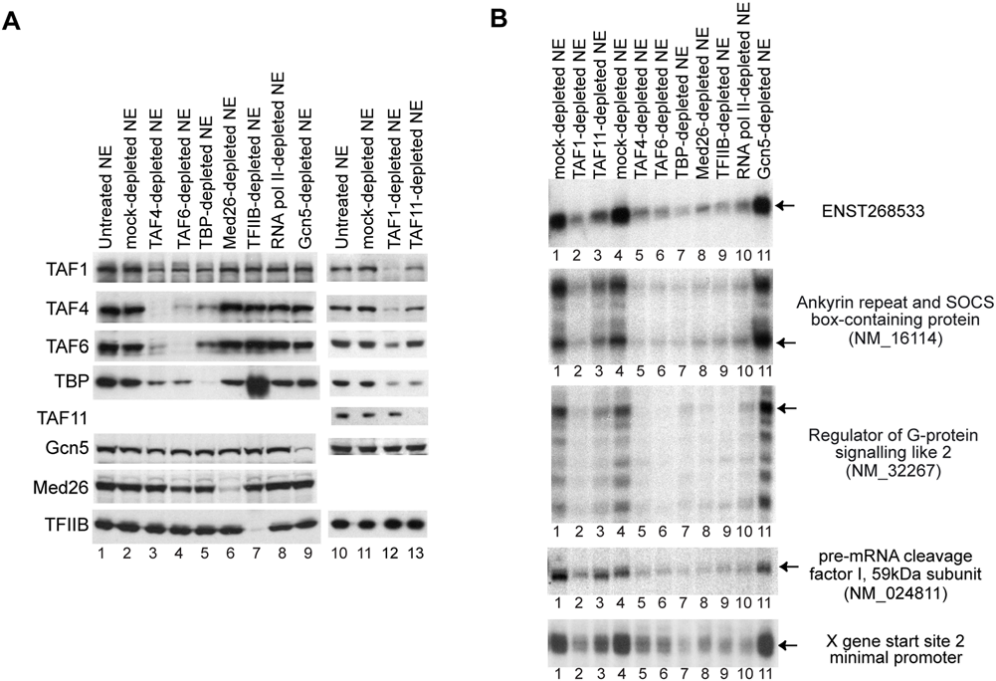
XCPE1 and XCPE2-containing cellular promoters show the same GTF requirements as HBV X gene promoter. (A) Western blot analyses of depleted NEs. HeLa NE was immunodepleted as indicated and the levels of target factors as well as other GTFs were examined by western blotting. (B) *In vitro* transcription assays of the immunodepleted NEs. Transcription activities for the XCPE2-containing promoters (ENST268533, NM_16114, and NM_32267), an XCPE1-containing promoter (NM_024811), and the X gene Start site 2 minimal promoter were examined. Arrows show the transcripts starting at the positions expected to be driven by XCPE2 or XCPE1.

We examined the GTF requirements for XCPE1- and XCPE2-containing cellular promoters. HeLa cell NE was immunodepleted as in the previous experiments and transcription activity for XCPE1 and XCPE2 cellular promoters was analyzed. To enhance levels of TAF1- and TAF11-depletion, we treated NE with TAF1 and TAF11 antibodies twice. The western blot analyses of antibody treatments are shown in Fig. 9A, and the transcription activity assays with the depleted NEs are shown in Fig. 9B. As a control, we also carried out *in vitro* transcription of the X gene Start site 2 minimal promoter (pX1020-1032CAT) using the same depleted NEs. We found that the XCPE1- and XCPE2-driven transcription in the cellular promoters show very similar GTF requirements to those of the X promoter. Interestingly, many of the other TSSs in these promoters also showed the same patterns of GTF requirements, suggesting that there may be a common set of factors utilized by dispersed promoters found in CpG islands.

### TFIIB, TBP, RNA pol II, TFIIF, and mediator are not sufficient for sequence-specific promoter recognition for XCPE2-containing promoters

**Figure 10:**
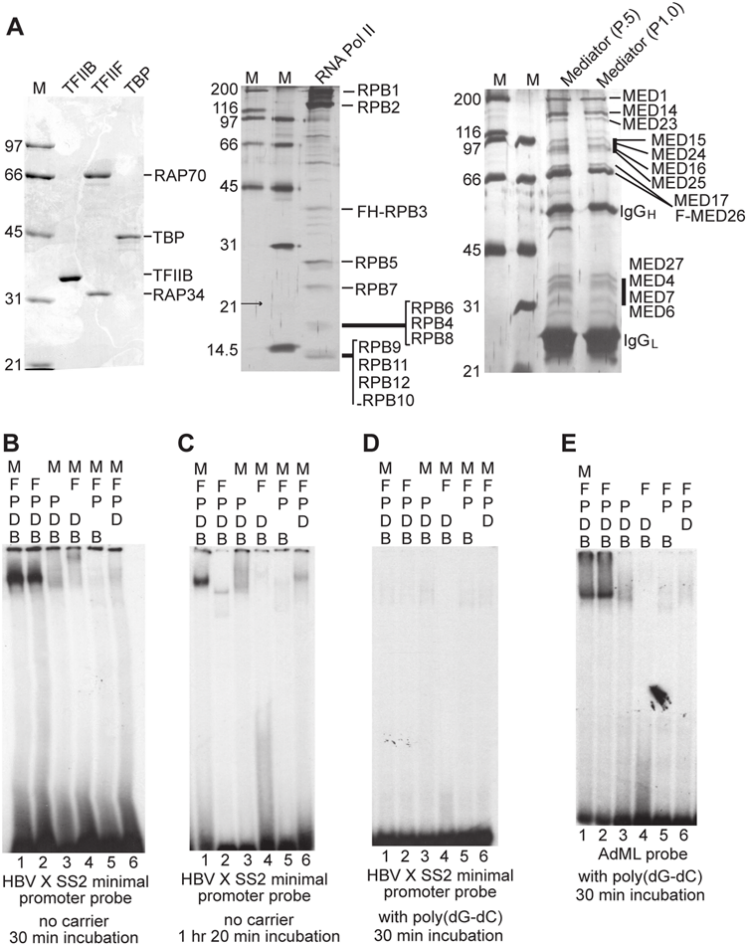
Sequence-nonspecific stable complex formation by TBP, TFIIB, RNA pol II, TFIIF, and mediator on X gene Start site 2 promoter. (A) SDS-PAGE analyses of purified factors. Purified recombinant TFIIB, TFIIF, and TBP were analyzed by Comassie staining. RNA pol II and mediator were analyzed by silver staining. Bands are labeled by comparing the previously published patterns. The mediator complexes shown are bound to M2 beads, from which they were eluted with FLAG peptides and used for EMSA. (B) EMSA with the X gene Start site 2 minimal promoter probe. The ^32^P-labeled probe was mixed with indicated factors without carrier DNA. B, TFIIB; D, TBP; P, RNA pol II; F, TFIIF; and M, mediator. The mixtures were incubated for 30 min before electrophoresis as described in Materials and Methods. The experiments shown were performed using the mediator complex from the phosphocellulose fraction P1.0, but the complexes from the P.5 fraction showed the same results. (C) XCPE2 DNA and TFIIB/TBP/Pol II/TFIIF form an unstable complex. The same EMSA as is shown in Fig. 10B was performed except that the binding mixtures were incubated for 1 hr 20 min before electrophoresis. (D) The XCPE2 DNA/TFIIB/TBP/Pol II/TFIIF/mediator complex was not observed in the presence of poly(dG-dG)· poly(dG-dC). (E) EMSA showing TFIIB/TBP/Pol II/TFIIF complex formation on the adenovirus major late (AdML) promoter. The binding mixtures were incubated for 30 min before electrophoresis. The same results were obtained when the mixtures were incubated for a longer period (2 hrs, data not shown).

Since we have found that transcription from XCPE2-containing promoters requires at least RNA pol II, TBP, mediator, and TFIIB (Figs. 3, 7, 8, and 9), we wanted to know whether these are sufficient for XCPE2 core promoter recognition. Some of the previous studies on Inr-containing promoters have shown that TBP (or TFIID), TFIIB, RNA pol II, and TFIIF could form a stable complex (DBPolF complex) at the core promoters in an Inr sequence-dependent manner [30], [31]. Therefore, we additionally purified RNA pol II and TFIIF and conducted EMSAs (electrophoretic mobility-shift assays) with ^32^P-labeled probe containing the X gene Start site 2 minimal promoter. As a control, we conducted EMSAs using ^32^P-labeled adenovirus major late (AdML) core promoter region (−38 to +13) that has previously been used to detect stable preinitiation complex formation [32]. Fig. 10A shows our purified TFIIB, TFIIF, TBP, RNA pol II, and mediator proteins, and Fig. 10B–E shows the EMSAs. We found that when all of the factors were mixed with the X gene Start site 2 promoter DNA, we could detect a stable discrete band (Fig. 10B, lane 1 and Fig. 10C, lane 1). A similar band was detected without the mediator (Fig. 10B, lane 2), but it often disappeared if the mixture was incubated for a longer time before the electrophoresis (An example is shown in Fig. 10C, lane 2). If any of the other factors was removed from the mixture, the band was not detected (Fig. 10B, lanes 3–6). Therefore, this specific combination of GTFs may have activity to bind XCPE2-containing promoter DNAs. However, if we added poly(dG-dC)·poly(dG-dC) to the EMSA binding reactions, this factor-specifically formed DNA-protein complex was not detected (Fig. 10D). Therefore this XCPE2 DNA-protein complex we observed did not appear to be formed sequence-specifically. In the control EMSAs using the AdML probe, a stable DNA protein complex was detected in the presence of TBP, TFIIB, RNA pol II, and TFIIF (DBPolF) with or without mediator, and was resistant to poly(dG-dC)·poly(dG-dC) (Fig. 10E, lanes 1 and 2) as expected. This control EMSA, combined with our transcriptional activity assays with the purified factors (Figs. 4, 5, and 7), indicates that our purified factors are functional and therefore the lack of sequence-specific XCPE2 recognition was not due to inactivity of the purified factors. Taken together, we could detect a stable protein-DNA complex formed on the XCPE2-containing X promoter DNA with TBP, TFIIB, RNA pol II, TFIIF, and mediator, but this combination of GTFs is still not sufficient for sequence-specific promoter recognition that would explain site-specific transcriptional initiation by XCPE2. Therefore, additional factors, such as other previously known GTFs and/or cofactors, must be required for specific transcriptional initiation from XCPE2-containing promoters. This result suggests that the mechanism of XCPE2 promoter recognition is different from those previously described for a class of Inr-containing TATA-less promoters.

## Discussion

### A new core promoter element XCPE2 that drives transcription from TATA-less promoters containing multiple transcriptional starts sites

In this study, we identified a new core promoter element XCPE2 that drives transcription from the second TSS of the HBV X gene. XCPE2 also appears to drive transcription typically from one of the TSSs present in human promoters that show clusters of TSSs. It is interesting to find that there are functional similarities between XCPE2 and our previously identified core promoter element XCPE1. First, both XCPE1 and XCPE2 are located around the start sites (−8∼+2 for XCPE1 and −9∼+2 for XCPE2). Second, the XCPE1 and XCPE2 sequences are mainly found in TATA-less promoters that often show multiple start sites. Finally, transcription from both start sites 1 and 2 displays the same or very similar GTF requirements. However, there are also some differences between XCPE1 and XCPE2; first, their consensus sequences are different (DSGYGGRASM vs. VCYCRTTRCMY), and second, XCPE1 requires activator-binding site(s) to show detectable transcription but XCPE2 can show a detectable level of transcription by itself.

Multiple studies have demonstrated the presence of enhancer-promoter specificity, i.e., different core promoters exhibit different and selective responses to enhancers, indicating that the combination of a specific promoter and enhancers determines the transcriptional regulation pattern for each gene [33]–[36]. The molecular basis of enhancer-promoter specificity is not completely clear, but one of the potential explanations may be that different core promoters utilize different sets of GTFs and cofactors [37] so that the regulatory signals can be differently transmitted, received, and interpreted. Therefore, determination of the complete set of factors that contribute to transcription of XCPE1- or XCPE2-driven promoters is a challenging task but appears critical to further our understanding of transcription mechanisms of a large number of TATA-less dispersed promoters.

### Mechanisms of transcription from XCPE1-and XCPE2-containing promoters are different from previously described mechanisms

To gain insights into mechanisms of XCPE1-and XCPE2-driven transcription, we examined requirements of several GTFs. Our immunodepletion and add-back experiments suggest that transcription from XCPE1- and XCPE2-containing promoters requires at least TFIIB, TFIID (or free TBP), RNA pol II and the mediator complex present in the nuclear extracts but not Gcn5. The observation that XCPE1 and XCPE2 could drive transcription without TAFs indicates that mechanisms for XCPE1- and XCPE2-containing promoters are different from the TFIID-dependent mechanisms described for the Inr- and DPE-containing promoters [18], [19], [38]. The observation that XCPE1- and XCPE2-driven transcription could occur without Gcn5 means that the mechanisms for XCPE1- and XCPE2-driven transcription are also different from the one described for yeast TATA-containing promoters using free TBP and SAGA (Spt-Ada-Gcn5) complex [23] or the one described for TATA-less, Inr-containing promoter using TFTC (TBP-free TAF_II_-containing complex) [14].

Previous studies have reported that the mediator complex purified from one of the phosphocellulose fractions (the 0.85 M KCl fraction) plays a role in supporting basal transcription [28], [29]. However, these studies clearly showed requirement of mediator for only either basal transcription from TATA-containing promoters or activated transcription from TATA-less Inr-containing promoters, and it was not clear whether mediator was required for basal transcription of their TATA-less promoters because the level of basal transcription from the promoter was almost background. In our previous study on XCPE1, we were also unable to discriminate whether mediator was required for basal transcription or activated transcription because XCPE1 was activator-dependent core promoter [6]. In this study, since the XCPE2 can drive a basal transcription by itself that is clearly distinguishable from background, we were able to clearly show an example of mediator requirement for basal transcription from a class of TATA-less promoters, i.e., XCPE2-containing promoters.

### TAF-free transcription

In this study, immunodepletion analyses showed that transcription from XCPE1- or XCPE2-containing promoters use at least RNA Pol II, TFIIB, mediator, and TFIID present in the NE as critical factors. However, we also showed that these promoters can use free TBP instead of TFIID if free TBP is available. In mammalian cells, it remains unclear exactly how much and in what conditions free TBP, the complete TFIID complex, and the TFIID sub-complexes exist. However, TAF-free transcription may also be utilized in mammalian cells because it has been shown that yeast cells utilize a free form of TBP for transcription of TATA-containing promoters by cooperation with the SAGA complex [23], [39]. In addition, previous *in vitro* reconstitution studies on TATA-containing promoters using HeLa cell extracts showed that TAFs are not absolutely required for activated transcription depending on core-promoter sequences around the TSSs, indicating that there may be alternative transcriptional initiation pathways which is TAF-independent [40], [41]. It would be interesting to compare GTF requirements for such TAF-free transcription from TATA-containing promoters and those for transcription from XCPE1/XCPE2 promoters.

Levels of TAFs in mammalian cells may substantially change according to the cell cycle phases or physiological conditions etc., consequently, the pool of TFIID in the cells may include certain amounts of sub-complexes and free TBP. If the levels of TAFs become low, the XCPE1 and XCPE2 classes of TATA-less genes would be continuously expressed, while expression of other TAF-dependent genes would be reduced. In higher eukaryotes, transcription mechanisms using a more diverse set of core promoter structures and different GTFs utilization might have evolved to enable the more complex and flexible regulation required for the multi-cellular environment. In order to further clarify mechanisms of transcriptional regulation for XCPE1 and XCPE2-containing promoters, our next goals will be to complete determination of factor requirements for transcription from XCPE1 and XCPE2 promoters by *in vitro* reconstitution either in the presence or absence of TAFs, and to identify XCPE1 and XCPE2 recognition factors. It will be interesting to also find what DNA sequences drive transcription from other TSSs in the XCPE1 and XCPE2-containing promoters or other multiple TSS-containing dispersed promoters. Information on what sub-fractions of TFIID are present *in vivo* will be also critical.

## Materials and Methods

### Plasmids

The HBV X gene DNAs are derived from a HBV strain (subtype adr) [42]. Nucleotide position no.1 (nt1) of this strain corresponds to nt127 of the strain whose EcoRI site is designated as nt1. The wild-type CAT (chloramphenicol acetyltransferase) reporter plasmid containing the minimal promoter for Start site 2 of the HBV X gene mRNA has been described [6]. For extensive point mutagenesis of the X core promoter 2, oligonucleotides containing mutated X core promoter 2 sequences were synthesized, annealed, and ligated into a CAT reporter plasmid pSV00CAT [43]. A control *in vitro* transcription template Sp1-TATA which contains multiple Sp1-binding sites and a TATA box from adenovirus E1B promoter has been described [24]. XCPE2-containing human promoter regions covering from about 500 bp upstream to about 50–100 bp downstream of XCPE2 sequences were cloned from Huh-7 hepatocellular carcinoma cell genomic DNA by PCR, and cloned into pSV00CAT. Subsequently, mutations of XCPE2 sequences were introduced by the Quick Change procedure (Stratagene). DNA sequences of all the clones (the wild-type and mutated promoters) were verified by DNA sequencing.

### Immunodepletion and *in vitro* transcription

The anti-TBP and anti-MED26 antibodies have been described [6]. Anti-Gcn5 and anti-TFIIB antibodies were from Santa Cruz Biotechnology, Inc. Anti-TAF11, anti-TAF6, and anti-RNA polymerase II (8WG16) were purchased from Abcam (ab50557), Bethyl laboratory, and Millipore, respectively. Anti-TAF1 was raised by immunizing rabbits with a TAF1 fragment (amino acids 1363–1638) which is a longer fragment than the fragment used to raise the previous anti-TAF1 [6]. Anti-human TAF4 was raised by immunizing rabbits with a TAF4 fragment (a.a. 1–197) and affinity-purified using the same protein fragment. Immunodepletion experiments and *in vitro* transcription assays were performed as described [6].

### Purification of TBP, TFIIB, TFIID, the mediator, TFIIF, and RNA pol II

Endogenous TFIID, FLAG-tagged MED26-containing mediator complex, and recombinant TBP were purified as described [6]. Recombinant untagged human TFIIB was expressed in E. *coli* and purified with Poros HS and Poros HE1 (Applied Biosystems) columns. Recombinant human TFIIF was purified as described previously [44]. RNA polymerase II was purified from HeLa cells that stably express FLAG-His-tagged Rpb3 as described [45].

### EMSA (electrophoretic mobility-shift assay)

Purified TFIIB (0.6 pmol), TBP (0.6 pmol), RNA pol II (0.3 pmol), TFIIF (1 pmol), and mediator (about 0.1 pmol or 200 ng) were mixed with 0.5–1.0 ng of ^32^P-labeled DNA probe in the presence of 20 mM Hepes, 10% Glycerol, 60 mM KCl, 4 mM MgCl_2_, and 4 mM DTT in a 15 µl reaction. In some reactions, 25 µg/ml poly(dG-dC)·poly(dG-dC) was added. After incubation at 30°C for 30 minutes or >1 hr, the mixture was analyzed by PAGE using a gel containing 4% acrylamide (37.5∶1), 2.5% Glycerol, and 0.5× TBE.

### Analysis of transcription from the XCPE2-containing promoter in transfected cells

HepG2 cells were transfected with CAT reporter plasmids driven by the XCPE2-containing promoters. Two days after transfection, poly(A)^+^ RNAs were purified and transcripts from the CAT reporter plasmids were analyzed by primer extension. For CAT reporter assays, whole cell extracts were prepared from transfected cells and CAT activity was measured as described [46].

## Supporting Information

Table S1 Human gene promoters that contain XCPE2 sequences around previously identified transcriptional start sites. A list of candidate human genes that utilize XCPE2
(0.06 MB XLS)

Figure S1 Activation of the X gene transcription from Start site 2 by addition of free TBP
(0.80 MB EPS)

## References

[1] Sandelin A, Carninci P, Lenhard B, Ponjavic J, Hayashizaki Y (2007). Mammalian RNA polymerase II core promoters: insights from genome-wide studies.. Nat Rev Genet.

[2] Bajic VB, Tan SL, Christoffels A, Schonbach C, Lipovich L (2006). Mice and men: their promoter properties.. PLoS Genet.

[3] FitzGerald PC, Shlyakhtenko A, Mir AA, Vinson C (2004). Clustering of DNA sequences in human promoters.. Genome Res.

[4] Frith MC, Valen E, Krogh A, Hayashizaki Y, Carninci P (2008). A code for transcription initiation in mammalian genomes.. Genome Res.

[5] Carninci P, Sandelin A, Lenhard B, Katayama S, Shimokawa K (2006). Genome-wide analysis of mammalian promoter architecture and evolution.. Nat Genet.

[6] Tokusumi Y, Ma Y, Song X, Jacobson RH, Takada S (2007). The new core promoter element XCPE1 (X Core Promoter Element 1) directs activator-, mediator-, and TATA-binding protein-dependent but TFIID-independent RNA polymerase II transcription from TATA-less promoters.. Mol Cell Biol.

[7] Smale ST, Kadonaga JT (2003). The RNA polymerase II core promoter.. Annu Rev Biochem.

[8] Butler JE, Kadonaga JT (2002). The RNA polymerase II core promoter: a key component in the regulation of gene expression.. Genes Dev.

[9] Lim CY, Santoso B, Boulay T, Dong E, Ohler U (2004). The MTE, a new core promoter element for transcription by RNA polymerase II.. Genes Dev.

[10] Ohler U, Liao GC, Niemann H, Rubin GM (2002). Computational analysis of core promoters in the Drosophila genome.. Genome Biol.

[11] Lee DH, Gershenzon N, Gupta M, Ioshikhes IP, Reinberg D (2005). Functional characterization of core promoter elements: the downstream core element is recognized by TAF1.. Mol Cell Biol.

[12] Suzuki Y, Tsunoda T, Sese J, Taira H, Mizushima-Sugano J (2001). Identification and characterization of the potential promoter regions of 1031 kinds of human genes.. Genome Res.

[13] Muller F, Demeny MA, Tora L (2007). New problems in RNA polymerase II transcription initiation: matching the diversity of core promoters with a variety of promoter recognition factors.. J Biol Chem.

[14] Wieczorek E, Brand M, Jacq X, Tora L (1998). Function of TAF(II)-containing complex without TBP in transcription by RNA polymerase II.. Nature.

[15] Martinez E, Kundu TK, Fu J, Roeder RG (1998). A human SPT3-TAFII31-GCN5-L acetylase complex distinct from transcription factor IID.. J Biol Chem.

[16] Hube F, Reverdiau P, Iochmann S, Gruel Y (2005). Improved PCR method for amplification of GC-rich DNA sequences.. Mol Biotechnol.

[17] Izawa M, Kitamur N, Odake N, Maki F, Kanehira K (2006). A rapid and simple transcriptional sequencing method for GC-rich DNA regions.. Jpn J Vet Res.

[18] Burke TW, Kadonaga JT (1997). The downstream core promoter element, DPE, is conserved from Drosophila to humans and is recognized by TAFII60 of Drosophila.. Genes Dev.

[19] Verrijzer CP, Chen JL, Yokomori K, Tjian R (1995). Binding of TAFs to core elements directs promoter selectivity by RNA polymerase II.. Cell.

[20] Wu PY, Ruhlmann C, Winston F, Schultz P (2004). Molecular architecture of the S. cerevisiae SAGA complex.. Mol Cell.

[21] Cavusoglu N, Brand M, Tora L, Van Dorsselaer A (2003). Novel subunits of the TATA binding protein free TAFII-containing transcription complex identified by matrix-assisted laser desorption/ionization-time of flight mass spectrometry following one-dimensional gel electrophoresis.. Proteomics.

[22] Wright KJ, Marr MT, Tjian R (2006). TAF4 nucleates a core subcomplex of TFIID and mediates activated transcription from a TATA-less promoter.. Proc Natl Acad Sci U S A.

[23] Sermwittayawong D, Tan S (2006). SAGA binds TBP via its Spt8 subunit in competition with DNA: implications for TBP recruitment.. Embo J.

[24] Ryu S, Zhou S, Ladurner AG, Tjian R (1999). The transcriptional cofactor complex CRSP is required for activity of the enhancer-binding protein Sp1.. Nature.

[25] Hayashida T, Sekiguchi T, Noguchi E, Sunamoto H, Ohba T (1994). The CCG1/TAFII250 gene is mutated in thermosensitive G1 mutants of the BHK21 cell line derived from golden hamster.. Gene.

[26] Hilton TL, Li Y, Dunphy EL, Wang EH (2005). TAF1 histone acetyltransferase activity in Sp1 activation of the cyclin D1 promoter.. Mol Cell Biol.

[27] Wang EH, Tjian R (1994). Promoter-selective transcriptional defect in cell cycle mutant ts13 rescued by hTAFII250.. Science.

[28] Mittler G, Kremmer E, Timmers HT, Meisterernst M (2001). Novel critical role of a human Mediator complex for basal RNA polymerase II transcription.. EMBO Rep.

[29] Wu SY, Zhou T, Chiang CM (2003). Human mediator enhances activator-facilitated recruitment of RNA polymerase II and promoter recognition by TATA-binding protein (TBP) independently of TBP-associated factors.. Mol Cell Biol.

[30] Weis L, Reinberg D (1997). Accurate positioning of RNA polymerase II on a natural TATA-less promoter is independent of TATA-binding-protein-associated factors and initiator-binding proteins.. Mol Cell Biol.

[31] Aso T, Conaway JW, Conaway RC (1994). Role of core promoter structure in assembly of the RNA polymerase II preinitiation complex. A common pathway for formation of preinitiation intermediates at many TATA and TATA-less promoters.. J Biol Chem.

[32] Ren D, Nedialkov YA, Li F, Xu D, Reimers S (2005). Spacing requirements for simultaneous recognition of the adenovirus major late promoter TATAAAAG box and initiator element.. Arch Biochem Biophys.

[33] Butler JE, Kadonaga JT (2001). Enhancer-promoter specificity mediated by DPE or TATA core promoter motifs.. Genes Dev.

[34] Ohtsuki S, Levine M, Cai HN (1998). Different core promoters possess distinct regulatory activities in the Drosophila embryo.. Genes Dev.

[35] Cheng JX, Floer M, Ononaji P, Bryant G, Ptashne M (2002). Responses of four yeast genes to changes in the transcriptional machinery are determined by their promoters.. Curr Biol.

[36] Li XY, Bhaumik SR, Zhu X, Li L, Shen WC (2002). Selective recruitment of TAFs by yeast upstream activating sequences. Implications for eukaryotic promoter structure.. Curr Biol.

[37] Thomas MC, Chiang CM (2006). The general transcription machinery and general cofactors.. Crit Rev Biochem Mol Biol.

[38] Burke TW, Kadonaga JT (1996). Drosophila TFIID binds to a conserved downstream basal promoter element that is present in many TATA-box-deficient promoters.. Genes Dev.

[39] Basehoar AD, Zanton SJ, Pugh BF (2004). Identification and distinct regulation of yeast TATA box-containing genes.. Cell.

[40] Oelgeschlager T, Tao Y, Kang YK, Roeder RG (1998). Transcription activation via enhanced preinitiation complex assembly in a human cell-free system lacking TAFIIs.. Mol Cell.

[41] Wu SY, Kershnar E, Chiang CM (1998). TAFII-independent activation mediated by human TBP in the presence of the positive cofactor PC4.. Embo J.

[42] Kobayashi M, Koike K (1984). Complete nucleotide sequence of hepatitis B virus DNA of subtype adr and its conserved gene organization.. Gene.

[43] Araki E, Shimada F, Shichiri M, Mori M, Ebina Y (1988). pSV00CAT: low background CAT plasmid.. Nucleic Acids Res.

[44] Wang BQ, Lei L, Burton ZF (1994). Importance of codon preference for production of human RAP74 and reconstitution of the RAP30/74 complex.. Protein Expr Purif.

[45] Hasegawa J, Endou M, Narita T, Yamada T, Yamaguchi Y (2003). A rapid purification method for human RNA polymerase II by two-step affinity chromatography.. J Biochem.

[46] Takada S, Kaneniwa N, Tsuchida N, Koike K (1996). Hepatitis B virus X gene expression is activated by X protein but repressed by p53 tumor suppressor gene product in the transient expression system.. Virology.

[47] Tokusumi Y, Zhou S, Takada S (2004). Nuclear respiratory factor 1 plays an essential role in transcriptional initiation from the hepatitis B virus x gene promoter.. J Virol.

